# Effects of PVA/TiO_2_ Composite Hydrogel-Modified PES Ultrafiltration Membranes on Antibiotic Removal Performance and Membrane Fouling Behavior

**DOI:** 10.3390/membranes16090293

**Published:** 2026-08-31

**Authors:** Mingyang Li, Bingyang Wang, Jianqiang Zhao, Lianghao Lv, Zhizhang Xu, Zhaoqian Xie, Xiaobo Wu, He Zhang, Guoliang Bai, Dingkun Lu

**Affiliations:** 1 Changjiang Basin Ecology and Environment Monitoring and Scientific Research Center, Changjiang Basin Ecology and Environment Administration, Ministry of Ecology and Environment, Wuhan 430010, China; 2 Hubei Key Laboratory of Intelligent Monitoring, Early Warning and Protection for Watershed Aquatic Ecology, Wuhan 430010, China; 3 Key Laboratory of Aquatic Organisms Monitoring and Assessment of the Changjiang Basin, Ministry of Ecology and Environment (Under Construction), Wuhan 430010, China; 4 Ganzhou Ecological Environmental Technology Service Center, Ganzhou 341000, China; 5 School of Resource and Environmental Engineering, Wuhan University of Technology, Wuhan 430070, China

**Keywords:** PES ultrafiltration membrane, PVA/TiO_2_ composite hydrogel, antibiotics, antibiotic resistance genes, membrane fouling

## Abstract

To improve the hydrophilicity and antibiotic retention of conventional polyethersulfone (PES) ultrafiltration membranes, a PVA/TiO_2_ composite hydrogel-modified PES membrane was prepared by hydrogel coating and in situ TiO_2_ embedding. Its removal of common antibiotics and antibiotic resistance genes (ARGs), as well as fouling behavior, was evaluated under different environmental conditions. The modified membrane formed an approximately 5 μm gel layer, showed reduced surface roughness, and increased pure-water flux by about 5.0%. It removed four antibiotics more effectively than the pristine membrane, with the greatest improvement for ofloxacin. Environmental conditions strongly affected antibiotic removal but had limited effects on ARG reduction. Low pH favored sulfamethoxazole, tetracycline, and ofloxacin removal, while 5 μm particles increased sulfamethoxazole and tetracycline removal by 27.0% and 22.0%, respectively. Hermia model fitting suggested predominantly standard blocking-type hydraulic behavior under the tested pH conditions and complete blocking-type behavior in the presence of HA or particles. These results show that PVA/TiO_2_ modification improves PES membrane hydrophilicity and antibiotic removal, although fouling under complex conditions remains important.

## 1. Introduction

Antibiotics are widely used in medical treatment, livestock and poultry farming, and aquaculture [1]. Because some antibiotics cannot be fully absorbed or utilized in organisms, they can continuously enter natural waters through domestic sewage, medical wastewater, aquaculture tailwater, and effluents from wastewater treatment plants, where they may persist for long periods [2]. This not only poses potential toxic effects on aquatic ecosystems, but also promotes the enrichment of antibiotic-resistant bacteria through continuous selective pressure, thereby facilitating the emergence, persistence, and dissemination of ARGs [3,4]. Compared with conventional organic pollutants, antibiotics and ARGs exhibit the dual characteristics of chemical pollution and biological risk; therefore, they have received increasing attention in assessments of drinking water safety and aquatic environmental health [5,6].

Existing studies on drinking water sources and treatment processes have shown that sulfonamides, macrolides, tetracyclines, and quinolones are typical antibiotics frequently detected in drinking water sources and treatment systems [7]. Among them, sulfamethoxazole (SMX), roxithromycin (ROX), tetracycline (TC), and ofloxacin (OFL), as representative compounds of these four antibiotic classes, have been widely used to investigate antibiotic removal behavior during water treatment [8]. Monitoring studies of full-scale drinking water treatment plants have reported that 17 of 30 target antibiotics were detected; among them, the detection frequencies of SMX, ROX, and OFL in influent water reached 100.0%, 87.5%, and 79.2%, respectively, with maximum concentrations of 42.5, 1.2, and 41.7 ng·L^−1^, respectively. The detection frequency of OFL in effluent water remained as high as 72.9%, indicating that some antibiotics can penetrate conventional treatment processes and remain in treated water [2]. In addition, tetracycline antibiotics have also been widely detected in surface water, groundwater, and drinking-water-related aquatic environments, and they are associated with relatively high ecological and resistance risks [9].

At present, conventional drinking water treatment processes are effective in removing particles, colloids, pathogenic microorganisms, and some macromolecular organic matter [10]. However, their capacity for simultaneous control of low-concentration, low-molecular-weight antibiotics and ARGs occurring in different forms remains insufficient [11]. Therefore, further investigation of their removal efficiencies during water treatment is of considerable significance. Ultrafiltration (UF) has been widely applied in advanced drinking water treatment because of its operational stability, relatively low energy consumption, and effective retention of particles, colloids, microorganisms, and some macromolecular organic matter [12,13]. However, typical antibiotics usually have molecular weights of only several hundred daltons, which are far below the molecular-weight cut-off of commonly used UF membranes; thus, they are difficult to remove effectively through size sieving alone [2,14]. ARGs may occur as intracellular DNA, extracellular free DNA, plasmid fragments, or forms adsorbed onto particles, colloids, and extracellular polymeric substances, and their transport and retention are likewise not governed solely by pore-size sieving [15]. Consequently, the removal of antibiotics and ARGs during UF is usually controlled by multiple mechanisms, including membrane-surface adsorption, intrapore retention, electrostatic interactions, hydrogen bonding, and auxiliary retention by fouling layers [2,16]. Therefore, membrane hydrophilicity, roughness, pore structure, and interfacial chemical properties are key factors affecting the removal of antibiotics and ARGs.

Polyethersulfone (PES) membranes have high mechanical strength, good thermal and chemical stability, and excellent film-forming properties, making them commonly used polymeric membrane materials in UF processes [17]. However, the PES molecular structure contains aromatic rings and sulfone-containing backbone structures, resulting in insufficient surface hydrophilicity. During filtration of complex water matrices, PES membranes are prone to organic adsorption, colloidal deposition, and pore blocking, which lead to flux decline and aggravated membrane fouling [18]. In particular, in waters containing antibiotics, natural organic matter, and particles, contaminant removal and membrane fouling processes are often coupled [19]. Therefore, improving membrane-surface hydrophilicity, optimizing pore structure, and enhancing interfacial interactions with target contaminants while maintaining the basic separation performance of PES membranes are critical for improving the removal of antibiotics and ARGs by PES membranes [20].

Constructing a hydrophilic functional layer is an effective strategy for improving the interfacial properties of PES membranes [21]. Polyvinyl alcohol (PVA) is rich in hydroxyl groups and can serve as the main film-forming component of a hydrogel layer, forming a continuous hydrophilic network and a stable hydration layer on the membrane surface, thereby reducing contaminant adhesion and promoting water transport [22]. Chitosan contains abundant amino and hydroxyl groups; it can not only improve the crosslinking stability of the hydrogel layer, but also provide interaction sites for contaminants such as antibiotics and DNA through hydrogen bonding, electrostatic interactions, or complexation [23]. TiO_2_ mainly acts as an inorganic hydrophilic filler and structural-regulation component. Its abundant surface hydroxyl groups help improve membrane-surface polarity and hydrophilicity, while its incorporation into the hydrogel network can regulate membrane-surface structure and interfacial properties [24]. Therefore, PVA, chitosan, and TiO_2_ respectively contribute to hydrophilic network construction, functional-site introduction, and interfacial-structure regulation. Their combination is expected to form an organic–inorganic composite hydrogel layer with hydrophilicity, stability, and interfacial-regulation capability.

Based on the above understanding and remaining problems, this study addresses the limited ability of PES UF membranes to remove typical antibiotics and ARGs and their susceptibility to fouling. A PES/PVA/TiO_2_-100 modified membrane was prepared by combining PVA/chitosan hydrogel coating with in situ TiO_2_ embedding. Specifically, this study: (i) characterized the effects of the modified layer on membrane surface morphology, functional-group composition, pore structure, hydrophilicity, and baseline hydraulic performance using scanning electron microscopy (SEM), atomic force microscopy (AFM), attenuated total reflection Fourier transform infrared spectroscopy (FTIR-ATR), and pore-size distribution analysis; (ii) compared the removal efficiencies of PES-100 and PES/PVA/TiO_2_-100 for SMX, ROX, TC, OFL, and target ARGs; (iii) further examined contaminant attenuation and flux variation in the modified membrane under complex water-quality conditions using pH, humic acid (HA), and particulate matter as environmental factors; and (iv) analyzed membrane fouling characteristics by combining FTIR-ATR characterization of fouled membranes with Hermia model fitting. This work aims to elucidate the effects of PVA/TiO_2_ composite hydrogel modification on the structure and separation performance of PES UF membranes, clarify the influence of complex water-quality conditions on antibiotic and ARG removal and membrane fouling behavior, and provide a reference for the coordinated control of antibiotics and ARGs and the application assessment of modified UF membranes.

## 2. Materials and Methods

### 2.1. Experimental Materials and Simulated Water Samples

The base membrane used in this study was a commercially available flat-sheet PES ultrafiltration membrane. PES-100, with a molecular weight cut-off of 100 kDa, was used as the base membrane for modification and as the unmodified control. The main reagents used for membrane modification included polyvinyl alcohol (PVA), chitosan, glutaraldehyde, methanol, acetic acid, and ammonium hexafluorotitanate ((NH_4_)_2_TiF_6_). In the water-matrix regulation experiments, HA was used as a heterogeneous surrogate for natural organic matter, while kaolin particles were used to simulate inorganic suspended particles in water [25]. The effects of HA were evaluated at the bulk matrix level based on the applied HA concentration.

The target antibiotics included sulfamethoxazole (SMX), roxithromycin (ROX), tetracycline (TC), and ofloxacin (OFL), representing sulfonamide, macrolide, tetracycline, and quinolone antibiotics, respectively. These compounds were selected to evaluate the removal efficiencies of the PES base membrane and the modified membrane for typical antibiotics from different classes [8]. Individual antibiotic stock solutions were prepared in methanol and stored at −18 °C in the dark. Before the filtration experiments, the four stock solutions were mixed and diluted with ultrapure water to prepare the simulated feed water. The nominal concentration of each antibiotic—SMX, ROX, TC, and OFL—in the simulated feed water was 200 μg·L^−1^. This concentration was selected to provide a controlled laboratory system for comparing membrane-retention behavior and to ensure reliable instrumental quantification after sample dilution.

The target ARGs included *sul1*, *sul2*, *ermA*, *ermB*, *qnrS*, *qnrD*, *tetM*, *tetO*, *tetW*, and *intI1*. These genes represented sulfonamide, macrolide, quinolone, and tetracycline resistance genes, as well as the integrase gene *intI1*, which can indicate the dissemination potential of ARGs. The 16S rDNA gene was used as an auxiliary reference gene [26]. ARG standards were prepared using standard plasmids. After copy-number conversion, the standards were mixed and diluted to prepare working standard solutions, which were then added to the simulated water samples so that the initial abundance of the target ARGs in the feed water was 1.0 × 10^8^ copies·mL^−1^. This setting was used to ensure the stability of qPCR quantification and to compare the reduction differences among different classes of ARGs.

### 2.2. Preparation of PVA/TiO_2_ Composite Hydrogel-Modified PES Membranes

PVA/TiO_2_ composite hydrogel-modified PES membranes were prepared by combining PVA/chitosan hydrogel coating with in situ TiO_2_ incorporation [22]. First, PES-100 membranes were immersed in absolute ethanol for at least 24 h, thoroughly rinsed with ultrapure water, and air-dried before modification.

To prepare the PVA/chitosan hydrogel, 0.40 g of PVA was dispersed in 150 mL of ultrapure water, gradually heated to 95 °C, and stirred at 80–100 rpm for 3 h until completely dissolved. Subsequently, 2.5 mL of 25% glutaraldehyde solution, 50 μL of methanol, and 75 μL of acetic acid were added. Separately, 0.40 g of chitosan was dissolved in 100 mL of 2% (*v*/*v*) acetic acid and ultrasonically degassed for 5 min. The chitosan solution was then thoroughly mixed with the PVA solution and maintained at 35 °C for 1 h to form the composite hydrogel. Based on the combined polymer mixture, the nominal concentrations of PVA and chitosan were both approximately 0.16 wt%.

The pretreated PES membrane was immersed in the composite hydrogel and coated at 30 °C and 150 rpm for 20 min to obtain PES/PVA-100. After thorough rinsing with ultrapure water, the membrane was immersed in 0.1 mol·L^−1^ (NH_4_)_2_TiF_6_ solution at pH 5 for 15 min to form TiO_2_ in situ within the hydrogel layer. The stated 0.1 mol·L^−1^ refers to the concentration of the (NH_4_)_2_TiF_6_ precursor solution rather than the final TiO_2_ loading in the membrane [27]. The membrane was then heat-treated at 30 °C for 60 min and immersed in ultrapure water for 24 h to remove unbound reagents, followed by thorough rinsing and storage until use [24].

The preparation conditions were selected based on preliminary screening of the PVA/chitosan content, coating conditions, (NH_4_)_2_TiF_6_ precursor conditions, and heat-treatment parameters. The unmodified PES membrane, the PVA/chitosan hydrogel-coated membrane, and the membrane further incorporated with TiO_2_ in situ were denoted as PES-100, PES/PVA-100, and PES/PVA/TiO_2_-100, respectively.

### 2.3. Ultrafiltration Experimental Design

#### 2.3.1. Ultrafiltration Setup and Basic Operation

Ultrafiltration experiments were conducted using a laboratory-scale constant-pressure ultrafiltration system consisting of a nitrogen gas source, pressure regulator, 400 mL ultrafiltration cell, magnetic stirrer, porous stainless-steel support, filtrate collection unit, and data-acquisition system. A schematic of the system and the principal operating parameters are provided in Figure S1 and Table S1, respectively, in the Supplementary Materials. The effective filtration area was 38.4 cm^2^, and the dead volume was approximately 1 mL. Before each experiment, the membrane coupon was fully wetted with ultrapure water and checked for integrity. After assembly, the system was tested with ultrapure water to confirm that no leakage occurred before the feed solution was introduced.

Each experiment used 400 mL of simulated feed water. Based on preliminary pressure screening of PES-100 at 0.05, 0.10, and 0.20 MPa, 0.10 MPa was selected to balance membrane flux and antibiotic retention, and the stirring speed was set at 200 rpm. Before filtrate collection, approximately 1 mL of dead volume was discharged, after which filtration time and filtrate volume were continuously recorded.

When the cumulative filtrate volume reached 100, 200, and 300 mL, 50 mL samples were collected and designated as Samples I, II, and III, respectively, for antibiotic analysis. After filtration, membrane coupons were collected for ARG extraction or fouled-membrane characterization, whereas the recorded filtration time and filtrate-volume data were used for subsequent flux analysis and fouling-model fitting. Filtrate samples were stored at 4 °C and analyzed as soon as possible.

Samples I, II, and III were collected sequentially during the same filtration run and represent stage-resolved samples at cumulative filtrate volumes of 100, 200, and 300 mL, respectively. They were used to characterize the evolution of apparent antibiotic retention during filtration rather than as three independent steady-state retention measurements.

#### 2.3.2. Comparative Experiments on Membrane Performance and Contaminant Removal Before and After Modification

Comparative filtration experiments were conducted using PES-100 and PES/PVA/TiO_2_-100 to evaluate changes in membrane flux, antibiotic retention, and membrane-associated ARG retention after composite modification. The operating conditions and sampling protocol followed those described in Section 2.3.1. Filtrate volume and filtration time were recorded throughout filtration; samples collected at cumulative filtrate volumes of 100, 200, and 300 mL were analyzed for SMX, ROX, TC, and OFL, and membrane coupons collected after filtration were used for target-ARG extraction and quantitative analysis.

#### 2.3.3. Removal Experiments with the Modified Membrane Under Different Environmental Conditions

All environmental-factor experiments were conducted using the PES/PVA/TiO_2_-100 modified membrane. The pH experiments were performed at pH 5, 7, and 9, representing mildly acidic, neutral, and mildly alkaline conditions, respectively, to examine pH-dependent antibiotic speciation, membrane–solute interactions, and membrane-associated ARG retention. HA was tested at 0, 5, and 10 mg·L^−1^ to evaluate the effects of HA concentration on contaminant retention and membrane fouling. Particle experiments were conducted without particles or with 5 or 10 μm kaolin at a fixed concentration of 2 mg·L^−1^ to evaluate the effect of particle size on contaminant retention and membrane-flux variation. The HA and particle experiments were conducted at pH 7.

All other operating conditions and sampling procedures followed those described in Section 2.3.1. Between experimental groups, the ultrafiltration system and related containers were thoroughly rinsed with ultrapure water to minimize cross-contamination.

#### 2.3.4. Fouled-Membrane Sampling and Fouling-Characteristic Analysis

Filtrate volume and filtration time were continuously recorded during filtration for flux-decline analysis and Hermia-model fitting [28]. After filtration, membrane coupons were collected and characterized by FTIR-ATR to examine changes in the exposure of the characteristic bands of the PVA/chitosan/TiO_2_ composite layer after filtration.

Membrane fouling was evaluated by integrating flux behavior, Hermia-model fitting, and post-filtration FTIR-ATR results. The Hermia models were used to characterize hydraulic flux-decline patterns, and the membrane fouling characteristics were evaluated by combining the flux decay curve and FTIR-ATR spectral changes.

### 2.4. Analytical Detection and Characterization Methods

#### 2.4.1. Antibiotic Detection

Before UPLC–MS/MS analysis, all samples were passed through 0.22 μm syringe filters to remove suspended particles and large colloidal aggregates. The filtrates were subsequently diluted tenfold. Thus, the nominal concentration of each antibiotic in the diluted feed sample was 20 μg·L^−1^. The measured concentrations of the diluted feed and permeate samples ranged from 5.11 to 17.80 μg·L^−1^ and were within the validated calibration range of 0.5–50 μg·L^−1^. The concentrations in the original water samples were calculated by applying the dilution factor of 10.

Antibiotics were detected using ultra-performance liquid chromatography-tandem mass spectrometry (UPLC-MS/MS). The target antibiotics included sulfamethoxazole (SMX), tetracycline (TC), ofloxacin (OFL), and roxithromycin (ROX). During sample analysis, quantitative determination was performed using an electrospray ionization source in positive-ion mode (ESI^+^) and multiple reaction monitoring (MRM).

Before analysis, the method was validated for linearity, limits of detection (LODs), limits of quantification (LOQs), and recovery. Seven calibration levels (0.5–50 μg·L^−1^), based on analyte/internal-standard peak-area ratios, yielded *R*^2^ values of 0.9970–0.9998. The LODs (S/N = 3) and LOQs (4 × LOD) were 0.04–0.09 and 0.16–0.36 ng/L, respectively. Spike recoveries at 5, 20, and 50 μg·L^−1^ were 59.5–102.8%, with RSDs below 11.3% (*n* = 6).

#### 2.4.2. Antibiotic Resistance Gene Detection

After filtration, membrane coupons were cut into small pieces, and DNA was extracted using a PowerSoil DNA Isolation Kit according to the manufacturer’s instructions. The extracted DNA was used as the template for qPCR analysis. Ten target genes (*qnrS*, *qnrD*, *tetM*, *tetO*, *tetW*, *sul1*, *sul2*, *ermA*, *ermB*, and *intI1*) were quantified, with 16S rDNA included as an auxiliary reference target [29]. Primer sequences, amplicon lengths, and annealing temperatures are listed in Table 1.

Antibiotic resistance genes were detected by real-time quantitative PCR (qPCR). The qPCR assays were performed using ChamQ Universal SYBR qPCR Master Mix (Vazyme, Nanjing, China). The total reaction volume was 20 μL, consisting of SYBR qPCR Master Mix, forward and reverse primers, DNA template, and ddH_2_O. The amplification program was as follows: initial denaturation at 95 °C for 30 s, followed by 39 cycles of denaturation at 95 °C for 10 s, annealing at the corresponding annealing temperature for 30 s, and extension at 72 °C for 30 s. A melting-curve analysis was then performed.

#### 2.4.3. Membrane Structure and Performance Characterization

Membrane surface and cross-sectional morphologies were characterized by scanning electron microscopy (SEM); cross-sectional specimens were fractured in liquid nitrogen and sputter-coated with gold before observation. Atomic force microscopy (AFM) was performed over 4 μm × 4 μm areas to obtain the maximum height difference (Rmax), average roughness (Ra), and root-mean-square roughness (Rms). Surface functional groups were analyzed by FTIR-ATR over 370–4000 cm^−1^ at a resolution of 0.44 cm^−1^. Nitrogen adsorption–desorption analysis was used to obtain pore-size distributions, which were interpreted together with the SEM and AFM results to evaluate structural changes after modification [30,31].

Membrane wettability was characterized by static water contact-angle measurements using 5 μL ultrapure-water droplets at 25 °C, with at least five measurements for each sample. Pure-water flux was used to evaluate baseline hydraulic performance and was calculated as described in Section 2.5.1. Interfacial stability of the modified layer was evaluated using an accelerated ultrasonication test. Membrane coupons were dried at 80 °C for 12 h and weighed before being ultrasonicated in 500 mL of ultrapure water for 2 h. After ultrasonication, the coupons were dried and weighed again, and changes in membrane mass and static water contact angle were compared to assess the integrity of the modified layer under the applied physical challenge.

### 2.5. Data Processing and Model Fitting

#### 2.5.1. Data Processing and Calculation

During filtration, filtrate volume and filtration time were recorded to calculate the specific filtrate volume, membrane flux, and flux decline [8,20]. To avoid symbol ambiguity, the specific filtrate volume was denoted as $V_{s}$ and calculated using Equation (1):

(1) $$V_{s}=\frac{V_{f}}{A}$$

where $V_{s}$ is the specific filtrate volume, m; $V_{f}$ is the cumulative filtrate volume, m^3^; and *A* is the effective membrane area, m^2^.

Membrane flux was calculated based on the increment in filtrate volume between adjacent recording points [32]. The average membrane flux during time interval t, denoted as $J_{t}$, was calculated using Equation (2):

(2) $$J_{t}=\frac{\Delta V_{f}}{A\cdot \Delta t}$$

where $J_{t}$ is the average membrane flux during a given filtration interval, m·s^−1^; $\Delta V_{f}$ is the increment in filtrate volume during that interval, m^3^; and Δt is the corresponding filtration time, s. The initial membrane flux $J_{0}$ was defined as the membrane flux during the initial filtration stage, and the final membrane flux was defined as the average membrane flux during the last recording interval before the end of filtration.

To compare membrane-flux decline under different water-quality conditions [33], normalized flux $J_{r}$ was used to characterize membrane-flux variation and was calculated using Equation (3):

(3) $$J_{r}=\frac{J_{t}}{J_{0}}$$

where $J_{r}$ is the normalized flux; $J_{t}$ is the average membrane flux during a given filtration interval, m·s^−1^; and $J_{0}$ is the initial membrane flux, m·s^−1^.

The removal efficiency of antibiotic i at sampling stage k, denoted as $R_{i,k}$, was calculated using Equation (4):

(4) $$R_{i,k}=\left(1-\frac{C_{p,i,k}}{C_{0,i}}\right)\times 100\%$$

where $R_{i,k}$ is the apparent retention of antibiotic i at sampling stage k, %; $C_{0,i}$ is the initial concentration of antibiotic i in the feed water, μg·L^−1^; and $C_{p,i,k}$ is the concentration of antibiotic i in permeate sample k, μg·L^−1^. Samples I, II, and III correspond to permeate samples collected at cumulative filtrate volumes of 100, 200, and 300 mL, respectively. The calculated values represent stage-resolved apparent retention based on the initial feed concentration and the permeate concentration measured at each sampling stage. Unless otherwise specified, the apparent retention determined for Sample III was used as the final-stage value for comparison among the experimental conditions. All antibiotic concentrations used in the calculation were obtained within the validated analytical range described in Section 2.4.1.

Membrane-associated ARG retention was characterized using the absolute copy numbers of target genes recovered from the membrane coupon after filtration and normalized to the filtered water volume. This indicator was used to compare ARG accumulation at the membrane interface under different experimental conditions. For target gene g, the absolute copy number detected on the membrane coupon was denoted as $N_{m,g}$, and the filtered water volume was denoted as $V_{0}$. The ARG retention abundance normalized by the filtered water volume, $C_{m,g}$, was calculated using Equation (5):

(5) $$C_{m,g}=\frac{N_{m,g}}{V_{0}}$$

where $C_{m,g}$ is the abundance of target gene g detected on the membrane coupon after normalization by filtered water volume, copies·mL^−1^; $N_{m,g}$ is the absolute copy number of target gene g detected on the membrane coupon after filtration, copies; and $V_{0}$ is the actual filtered water volume, mL.

The reduction efficiency of target gene g, $R_{g}$, was calculated using Equation (6):

(6) $$R_{g}=\frac{C_{m,g}}{C_{0,g}}\times 100\%$$

where $R_{g}$ is the reduction efficiency of target gene g, %; $C_{m,g}$ is the abundance of target gene g detected on the membrane coupon after normalization by filtered water volume, copies·mL^−1^; and $C_{0,g}$ is the initial abundance of target gene g in the influent, copies·mL^−1^. When the results were summarized by gene class, the $R_{g}$ values of target genes belonging to the same class were arithmetically averaged to obtain the average reduction efficiencies of SAs-ARGs, MCs-ARGs, FQs-ARGs, and TCs-ARGs.

The ${log}_{10}$-transformed membrane-retained ARG abundance was calculated using Equation (7):

(7) $$L_{g}=\log_{10}\left(C_{m,g}\right)$$

where $L_{g}$ is the ${log}_{10}$-transformed value of the abundance of target gene g detected on the membrane coupon. This value was used only to describe the order of magnitude of the absolute ARG abundance detected on the membrane coupon and was not equivalent to the conventional log removal value. When $C_{m,g}$ was below the detection limit or was not detected, log transformation was not performed, and the result could be reported according to the detection-limit or non-detection rule.

ARG standard plasmids were used for qPCR standard-curve construction and absolute quantification. The copy-number concentration of the standard plasmid, $N_{copy}$, was calculated using Equation (8):

(8) $$N_{copy}=\frac{C\times 10^{-9}\times N_{A}}{n\times 660}$$

where $N_{copy}$ is the copy-number concentration of the standard plasmid, copies·μL^−1^; *C* is the mass concentration of standard plasmid DNA, ng·μL^−1^; $N_{A}$ is Avogadro’s constant, taken as 6.02 × 10^23^ mol^−1^; *n* is the number of base pairs in the target fragment; and 660 is the average relative molecular mass of one base pair of double-stranded DNA, g·mol^−1^.

#### 2.5.2. Hermia Model Fitting for Hydraulic Fouling Characterization

The constant-pressure Hermia model was applied to the relationship between specific filtrate volume ($V_{s}$) and filtration time (*t*) to characterize the dominant hydraulic fouling pattern during filtration [34]. The four Hermia models represent complete blocking, standard blocking, intermediate blocking, and cake filtration, respectively, and were used to compare the predominant hydraulic flux-decline patterns under different water-matrix conditions.

The four models are expressed as:

(9) $$V_{s}=\frac{J_{0}}{K_{b}}\left(1-e^{-K_{b}t}\right)$$

(10) $$\frac{t}{V_{s}}=\frac{1}{J_{0}}+\frac{K_{s}}{2}t$$

(11) $$V_{s}=\frac{1}{K_{i}}\ln\left(1+K_{i}J_{0}t\right)$$

(12) $$\frac{t}{V_{s}}=\frac{1}{J_{0}}+\frac{K_{c}}{2}V_{s}$$

where t is filtration time, s; $V_{s}$ is the specific filtrate volume, m; $J_{0}$ is the initial membrane flux, m·s^−1^; $K_{b}$ is the complete pore-blocking constant, s^−1^; $K_{s}$ is the standard pore-blocking constant, m^−1^; $K_{i}$ is the intermediate pore-blocking constant, m^−1^; and $K_{c}$ is the cake-filtration constant, s·m^−2^. The isolated t on the right-hand side of Equation (10) still denotes filtration time and serves as the independent variable in the linear fitting of the standard pore-blocking model.

The goodness of fit was evaluated using the coefficient of determination $R^{2}$, calculated using Equation (13):

(13) $$R^{2}=1-\frac{\sum_{j=1}^{m}{\left(y_{j}-{\hat{y}}_{j}\right)}^{2}}{\sum_{j=1}^{m}{\left(y_{j}-\overset{-}{y}\right)}^{2}}$$

where $y_{j}$, ${\hat{y}}_{j}$, and $\overset{-}{y}$ are the measured, model-predicted, and mean measured values, respectively, and m is the number of data points. An $R^{2}$ value closer to 1 indicates a better fit. Hermia-model fitting was interpreted together with the corresponding flux-decline behavior and FTIR-ATR spectral changes to identify the predominant fouling characteristics under different water-matrix conditions.

## 3. Results and Discussion

### 3.1. Characterization of the Modified Membrane

PVA/chitosan hydrogel coating and in situ TiO_2_ embedding altered the surface structure and interfacial properties of the PES membrane, mainly as reflected by the formation of a composite modified layer, reduced surface roughness, adjusted pore structure, and enhanced hydrophilicity. The wettability, roughness, and chemical composition of the membrane surface can affect water transport, contaminant retention, and membrane fouling behavior [32,33]. Therefore, characterization of the morphology, functional groups, pore structure, and hydrophilicity of the modified membrane provides a structural basis for subsequent analysis of changes in filtration performance and contaminant removal.

**Figure 1:**
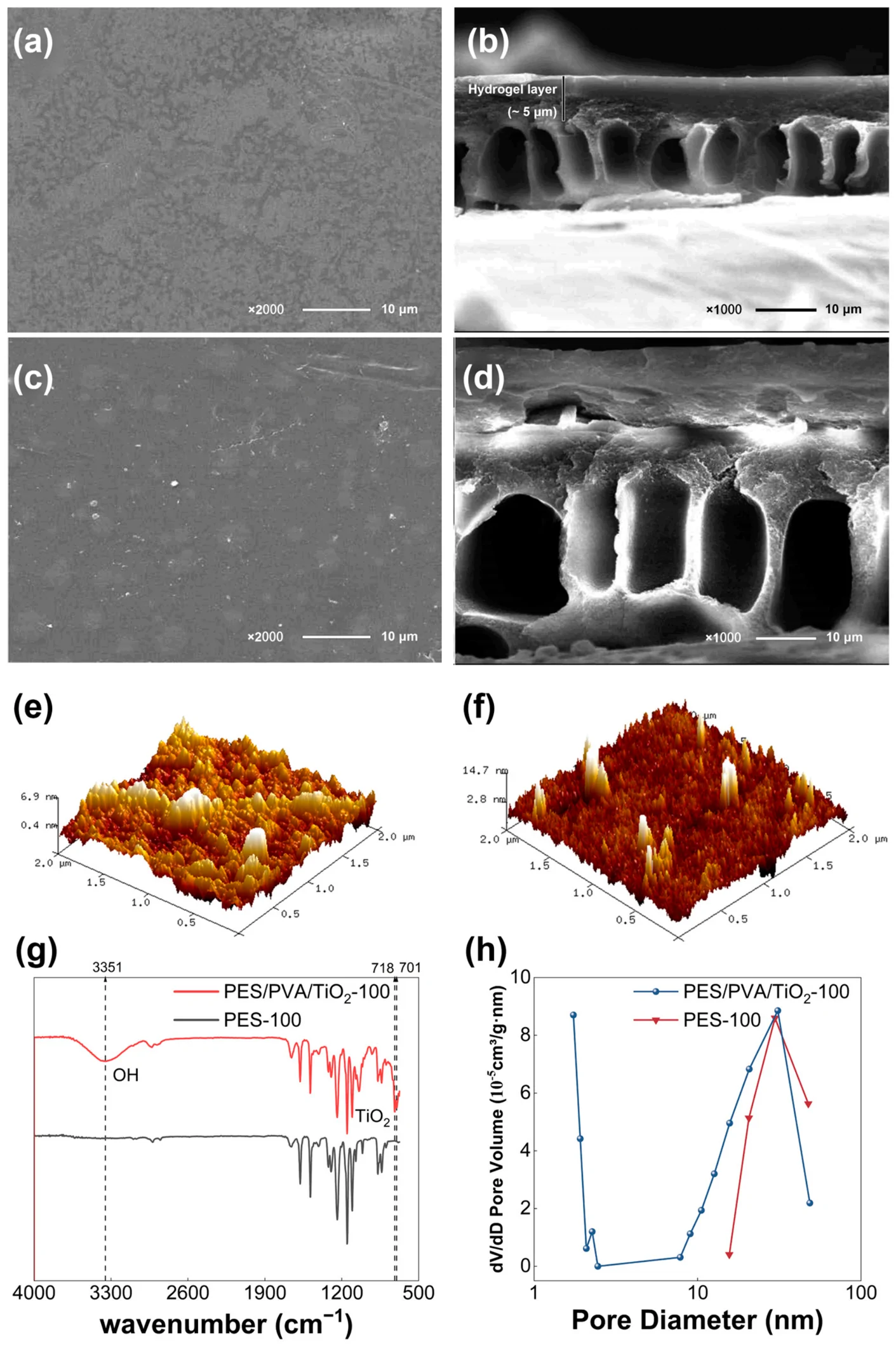
Characterization of PES/PVA/TiO_2_-100 and PES-100 membranes: (**a**,**c**) surface and (**b**,**d**) cross-sectional SEM images, where (**a**,**b**) show PES/PVA/TiO_2_-100 and (**c**,**d**) PES-100; (**e**,**f**) AFM images in the same order; (**g**) FTIR-ATR spectra; (**h**) pore-size distributions.

PVA/chitosan hydrogel coating and in situ TiO_2_ incorporation produced clear changes in the morphology, surface chemistry, pore-size distribution, and interfacial properties of PES-100 (Figure 1 and Table 2). SEM images (Figure 1a–d) showed that PES/PVA/TiO_2_-100 contained nanoparticle-rich regions with local aggregation rather than a uniformly distributed nanoscale particle layer, while the cross-sectional image revealed an approximately 5 μm-thick composite surface layer [35,36]. AFM analysis showed that Rmax, Ra, and Rms decreased from 20.486, 6.946, and 2.208 nm for PES-100 to 7.808, 2.344, and 0.873 nm for PES/PVA/TiO_2_-100, respectively, indicating substantially reduced surface roughness [37].

FTIR-ATR spectra (Figure 1g) showed an O–H band at 3150–3650 cm^−1^ and Ti–O bands at 718 and 701 cm^−1^ for PES/PVA/TiO_2_-100, supporting the incorporation of the PVA/chitosan hydrogel and TiO_2_-related structures into the composite layer [38,39,40]. The O–H and Ti–O bands provide characteristic spectral markers for the organic hydrogel component and TiO_2_-related structures, respectively. These diagnostic bands were therefore used in the subsequent comparison of membrane surfaces before and after filtration, where their retention, attenuation, or masking was used to evaluate the surface exposure of the modified layer under different filtration conditions.

The pore-size distribution (Figure 1h) also changed after modification, with a decreased contribution of pores around 50 nm and an increased contribution in the 2–4 nm range. These changes indicate redistribution and refinement of accessible pore sizes rather than a simple increase or decrease in overall membrane porosity. Pore-size distribution should therefore be interpreted together with membrane morphology and wettability when considering membrane structure–performance relationships [41]. Recent analysis of UF-membrane performance similarly identifies pore size, porosity, and contact angle as complementary rather than interchangeable membrane descriptors.

The static water contact angles of PES-100, PES/PVA-100, and PES/PVA/TiO_2_-100 were 94.5°, 70.1°, and 67.2°, respectively, while their pure-water fluxes were 2.16 × 10^−4^, 2.03 × 10^−4^, and 2.26 × 10^−4^ m·s^−1^. The PVA/chitosan hydrogel substantially improved membrane wettability but also introduced additional water-transport resistance, resulting in a slight decrease in pure-water flux for PES/PVA-100. Subsequent TiO_2_ incorporation further improved wettability and partly compensated for the resistance introduced by the hydrogel layer through interfacial and pore-structure regulation. Consequently, the pronounced improvement in hydrophilicity resulted in only an approximately 5% net increase in pure-water flux for PES/PVA/TiO_2_-100, reflecting the combined effects of improved interfacial wettability and additional structural resistance. Comparable hydrogel and TiO_2_-modified membrane studies also show that improved surface wettability does not translate directly into proportional flux enhancement because transport remains dependent on the structure and resistance of the modified layer [39].

After 2 h of accelerated ultrasonication, the material losses of PES/PVA-100 and PES/PVA/TiO_2_-100 were 0.19% and 0.24%, respectively, with no appreciable changes in their static water contact angles, indicating that the modified layers maintained interfacial integrity and surface wettability under the applied physical challenge.

### 3.2. Comparison of the Baseline Performance of the Modified Membrane and the Pristine PES Membrane

**Figure 2:**
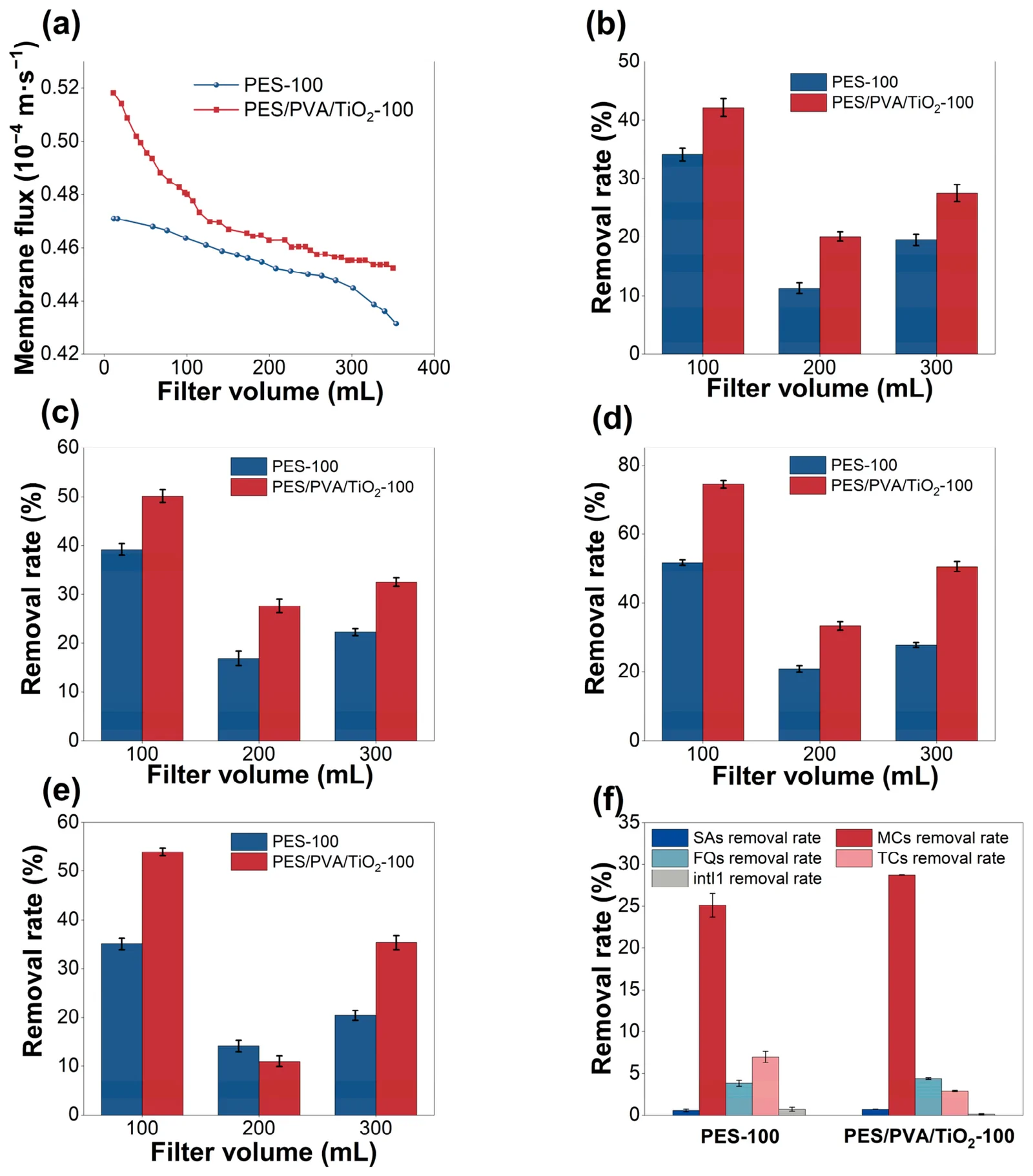
Membrane-flux variation and contaminant reduction by PES-100 and PES/PVA/TiO_2_-100 under baseline water-matrix conditions: (**a**) membrane flux; (**b**) SMX; (**c**) ROX; (**d**) TC; (**e**) OFL; (**f**) ARGs.

Under baseline water-matrix conditions, PES-100 and PES/PVA/TiO_2_-100 were compared in terms of membrane flux, antibiotic retention, and membrane-associated ARG retention (Figure 2). The initial and final fluxes of PES-100 were 4.71 × 10^−5^ and 4.31 × 10^−5^ m·s^−1^, respectively, whereas those of PES/PVA/TiO_2_-100 were 5.18 × 10^−5^ and 4.52 × 10^−5^ m·s^−1^, corresponding to increases of approximately 10.0% and 4.9%. These results were consistent with the improved membrane hydrophilicity described in Section 3.1 [42]. The hydrophilic hydroxyl and amino groups of the PVA/chitosan hydrogel and Ti–OH/Ti–O-related sites of TiO_2_ further increased membrane polarity and wettability [24], whereas the additional transport resistance introduced by the composite layer limited the overall flux enhancement.

Compared with PES-100, PES/PVA/TiO_2_-100 increased the final apparent retention of SMX, ROX, TC, and OFL by more than 10%, with the largest improvement observed for OFL (~14%; Figure 2b–e). Because the molecular weights of all four antibiotics are far below the 100 kDa MWCO, the enhanced retention after modification cannot be attributed primarily to size exclusion. Hydroxyl and amino groups in the PVA/chitosan layer and Ti–O-related polar sites provide a more interactive membrane interface, where surface adsorption, hydrogen bonding, electrostatic interactions, complexation, and intrapore retention may jointly contribute to the retention of low-molecular-weight antibiotics [37].

FTIR-ATR and water-contact-angle results confirmed that modification altered the chemical and interfacial properties of the membrane surface. The polar sites associated with PVA/chitosan and TiO_2_ may contribute to changes in local interfacial charge under different solution conditions; therefore, electrostatic interactions may participate in antibiotic retention. However, the chemically heterogeneous PVA/chitosan/TiO_2_ interface cannot be described solely by a single surface-charge parameter, and the observed retention should instead be interpreted as the combined result of multiple interfacial interactions [43]. The magnitude of improvement differed among the antibiotics. OFL is relatively polar under neutral conditions and contains functional groups capable of participating in hydrogen bonding, electrostatic interactions, and complexation [44], which may partly explain its larger increase after modification.

Compared with the antibiotics, the average membrane-associated ARG retention index increased by only approximately 5% for PES/PVA/TiO_2_-100 (Figure 2f). Standard plasmids were used as the ARG targets, and the index was calculated from target-gene copies recovered from the membrane coupons; it therefore represents plasmid-DNA accumulation and retention at the membrane interface rather than overall ARG removal calculated from feed and permeate concentrations. Membrane-DNA interactions and target-specific characteristics may contribute to the gene-dependent retention responses [8,45]. Overall, the composite modification enhanced the retention of the four low-molecular-weight antibiotics more strongly than the membrane-associated retention of the standard plasmid DNA.

### 3.3. Effects of Environmental Factors on the Removal Performance of the Modified Membrane

Under baseline water-matrix conditions, PES/PVA/TiO_2_-100 exhibited higher membrane flux and apparent antibiotic retention than PES-100. The effects of pH, HA, and particulate matter on membrane flux, stage-resolved apparent antibiotic retention, and membrane-associated ARG retention were subsequently evaluated.

Samples I, II, and III represent stage-resolved apparent retention at cumulative filtrate volumes of 100, 200, and 300 mL, respectively, rather than three independent steady-state retention values. Accordingly, non-monotonic variations among filtration stages should be interpreted in the context of evolving adsorption-site occupancy, membrane–solute interactions, and progressive foulant deposition during filtration.

#### 3.3.1. Effects of Different pH Conditions on Antibiotic and ARG Removal

**Figure 3:**
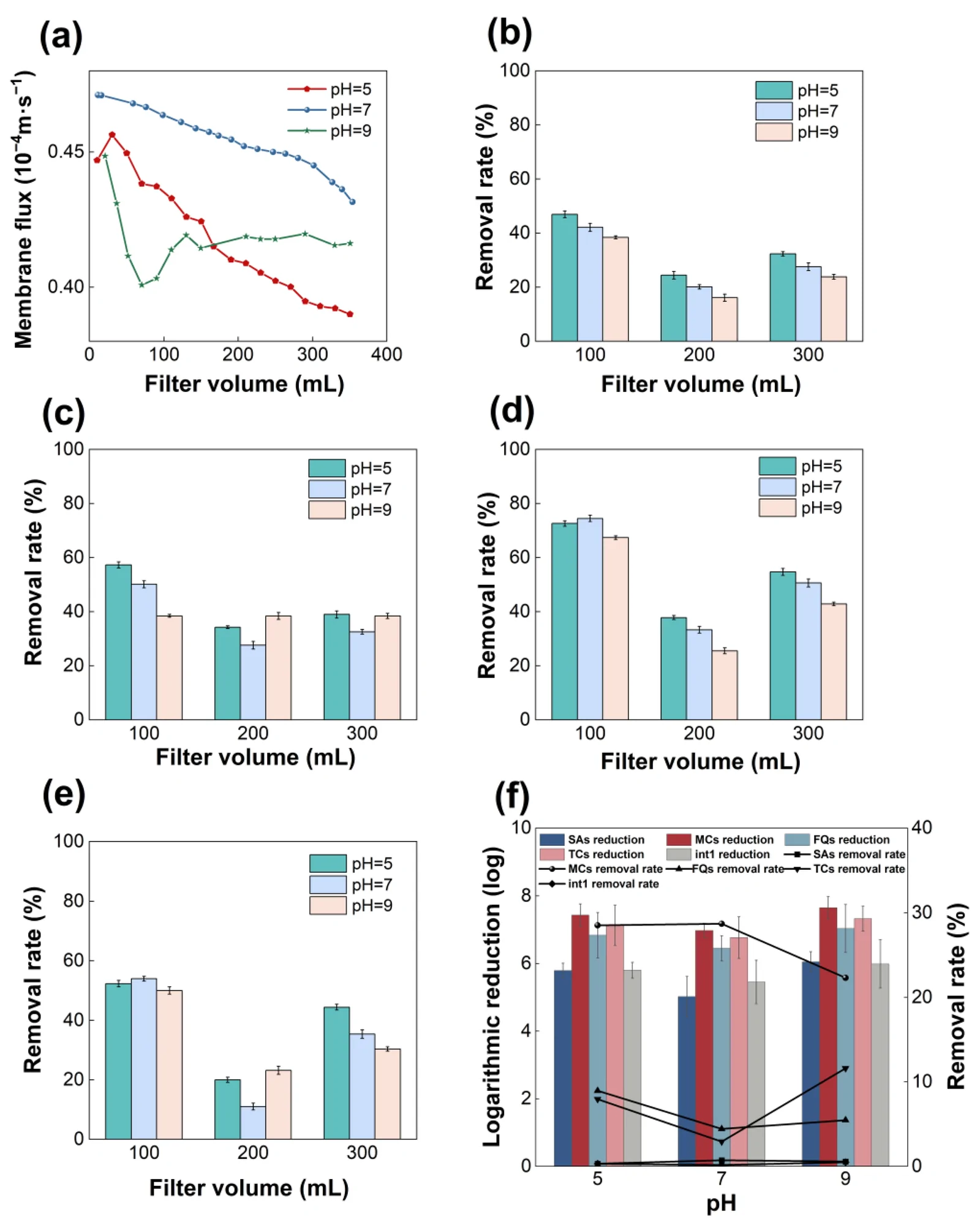
Membrane-flux variation and contaminant reduction by PES/PVA/TiO_2_-100 under different pH conditions: (**a**) membrane flux; (**b**) SMX; (**c**) ROX; (**d**) TC; (**e**) OFL; (**f**) ARGs.

pH can alter antibiotic ionization and membrane–solute interactions. As shown in Figure 3, SMX, TC, and OFL exhibited relatively clear pH-dependent responses, whereas ROX varied less over the investigated pH range of 5–9. The stage-dependent variations were interpreted within the sequential filtration framework described above. The discussion therefore focuses on the overall pH-associated response and uses Sample III as the final sampling stage for comparison among the tested pH conditions, without assigning the individual stage-to-stage fluctuations to a single process. Using Sample III as the final evaluation stage, SMX, TC, and OFL showed relatively high apparent retention at pH 5, with the final apparent retention of TC reaching 54.6%, indicating that acidic conditions favored the retention of some antibiotics in the present system [46].

The selected pH values of 5, 7, and 9 represent mildly acidic, neutral, and mildly alkaline conditions, respectively, and allow comparison of pH-dependent changes in antibiotic ionization and membrane interfacial properties. For the PVA/chitosan/TiO_2_ composite layer, changes in pH may alter the state of polar or ionizable interfacial groups, allowing electrostatic interactions to contribute to membrane–antibiotic interactions. However, because the modified layer constitutes a chemically heterogeneous composite interface, its pH response should not be assigned to a single surface-charge parameter but should instead be interpreted together with antibiotic speciation and other interfacial processes, including hydrogen bonding, complexation, and adsorption. SMX, TC, and OFL were more responsive to these pH-dependent interfacial changes, whereas ROX showed a comparatively weaker response.

Compared with antibiotics, ARGs showed inconsistent responses to pH changes. Under alkaline conditions, MCs-ARGs exhibited relatively better reductions, with the removal efficiencies of *ermA* and *ermB* reaching 19.0% and 25.6%, respectively. However, the removal efficiencies of SAs-ARGs and *intI1* remained low. Because ARGs can exist in multiple forms, such as intracellular DNA, extracellular DNA, or particle-associated carriers, their reduction is strongly affected by their occurrence forms [47]. Therefore, pH variation mainly resulted in differences among ARG classes rather than a uniform enhancement trend.

Overall, low-pH conditions favored the removal of SMX, TC, and OFL, but had limited promoting effects on ARG reduction. Because no additional HA or particulate matter was added in the pH experiments, changes in membrane flux were mainly associated with intrapore adsorption, local pore blocking, and related factors. This indicates that acid-base conditions not only affect contaminant removal, but also influence fouling behavior during membrane filtration [46].

#### 3.3.2. Effects of Different HA Concentrations on the Removal of Antibiotics and ARGs

**Figure 4:**
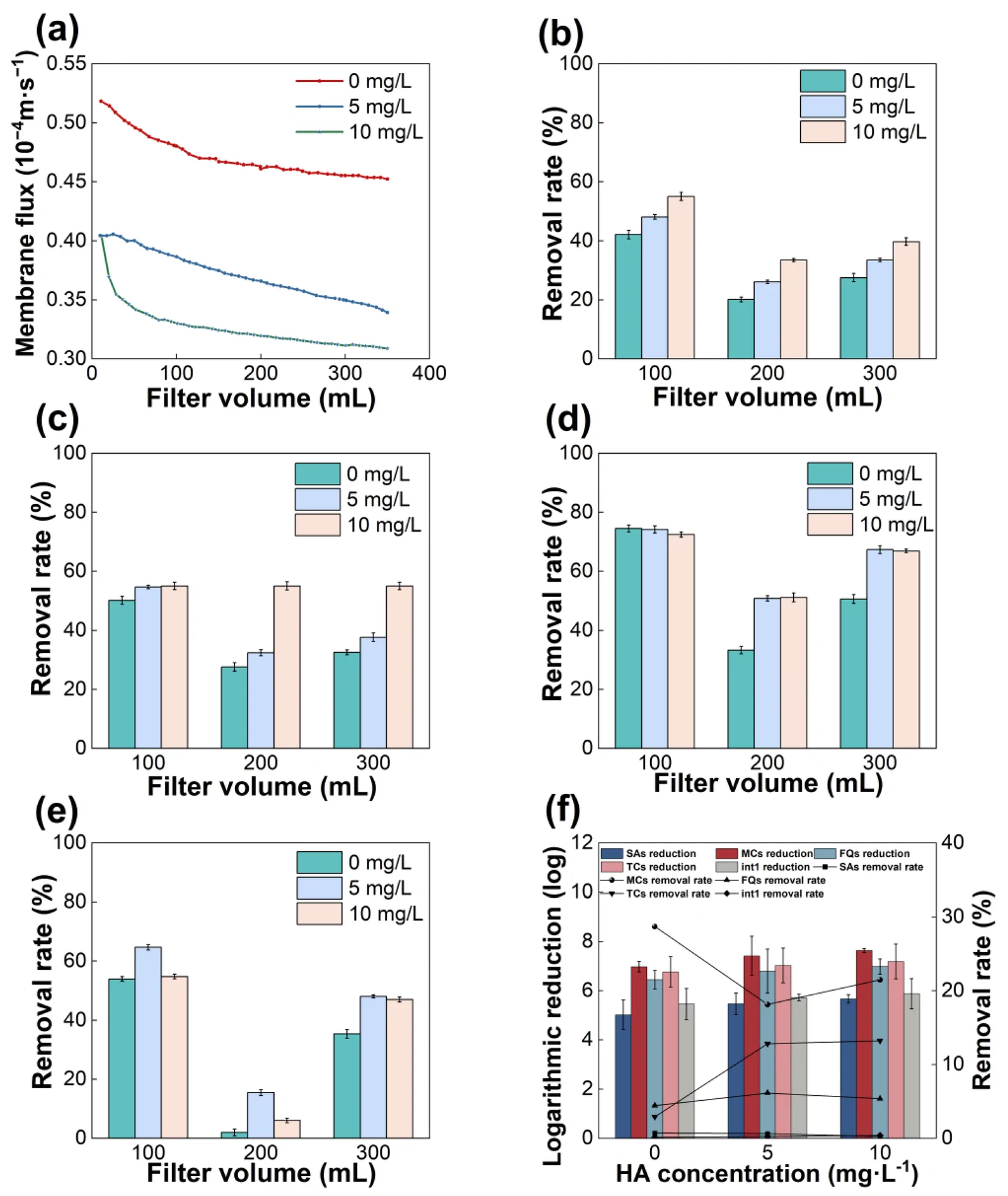
Membrane-flux variation and contaminant reduction by PES/PVA/TiO_2_-100 under different HA concentrations: (**a**) membrane flux; (**b**) SMX; (**c**) ROX; (**d**) TC; (**e**) OFL; (**f**) ARGs.

HA is an important representative of natural organic matter and can influence contaminant migration behavior and membrane filtration through adsorption, complexation, and membrane-surface deposition [48]. As shown in Figure 4, after HA addition, the membrane flux generally decreased, and the flux in the 10 mg·L^−1^ HA group was lower than that in the 5 mg·L^−1^ HA group and the control group. This indicates that HA increased membrane filtration resistance and aggravated membrane fouling.

HA markedly affected the stage-resolved apparent retention of the four antibiotics (Figure 4b–e). Samples I, II, and III were collected sequentially during the same filtration run and therefore reflect the evolution of the membrane–solute system at different filtration stages. The variations among the three samples were interpreted as combined stage-dependent responses during filtration rather than being assigned to any single process. Accordingly, the discussion focuses on the overall HA-associated response and uses Sample III as the final sampling stage for comparison. Compared with the HA-free condition, 5 mg·L^−1^ HA increased the final apparent retention of SMX, ROX, TC, and OFL by 6.0%, 5.1%, 16.8%, and 12.8%, respectively, with the largest increase observed for TC. The higher apparent retention observed in the presence of HA may reflect the combined contributions of size-dependent retention of HA-associated components, HA–antibiotic interactions, and HA accumulation at the membrane interface [49]. These processes were considered collectively when interpreting the overall HA-associated response. Increasing the HA concentration from 5 to 10 mg·L^−1^ did not produce a consistent further increase in the apparent retention of the four antibiotics, indicating concentration- and contaminant-dependent responses. Meanwhile, the greater flux decline at the higher HA concentration indicated that the changes in apparent retention were accompanied by increased filtration resistance.

In contrast, HA exerted a relatively limited and target-dependent effect on membrane-associated ARG retention (Figure 4f). At 5 and 10 mg·L^−1^ HA, the membrane-associated retention index of TCs-ARGs increased by 9.9% and 3.8%, respectively, relative to the HA-free condition, whereas the other ARG classes showed comparatively small changes. Because standard plasmids were used as the ARG targets, these results primarily represent changes in the relative accumulation and retention of plasmid DNA at the membrane interface in the presence of HA rather than the overall removal behavior of environmentally occurring ARGs in different forms.

Overall, the presence of HA was associated with higher apparent retention of some antibiotics, particularly TC, while simultaneously increasing filtration resistance. The observed retention response may reflect the combined effects of size-dependent retention of HA-associated components, HA-antibiotic interactions, and HA accumulation at the membrane interface. HA did not produce a consistent enhancement in membrane-associated ARG retention. The HA-containing system therefore exhibited a trade-off between apparent antibiotic retention and membrane-flux decline. The corresponding hydraulic fouling characteristics and membrane-surface spectral changes are further evaluated in Section 3.4 by integrating flux behavior, post-filtration FTIR-ATR, and Hermia-model fitting.

#### 3.3.3. Effects of Particulate-Matter Conditions on Antibiotic and Membrane-Associated ARG Retention

Particulate matter can act as an adsorption carrier to promote the transfer of antibiotics to the solid phase and can also form a particle-deposition layer on the membrane surface, thereby producing an additional retention effect [19]. On the other hand, particle deposition can increase filtration resistance and aggravate pore-mouth blocking. Therefore, differences in particle size may further affect the membrane flux and contaminant removal performance of PES/PVA/TiO_2_-100 by altering membrane-surface deposition behavior and the structure of the fouling layer.

**Figure 5:**
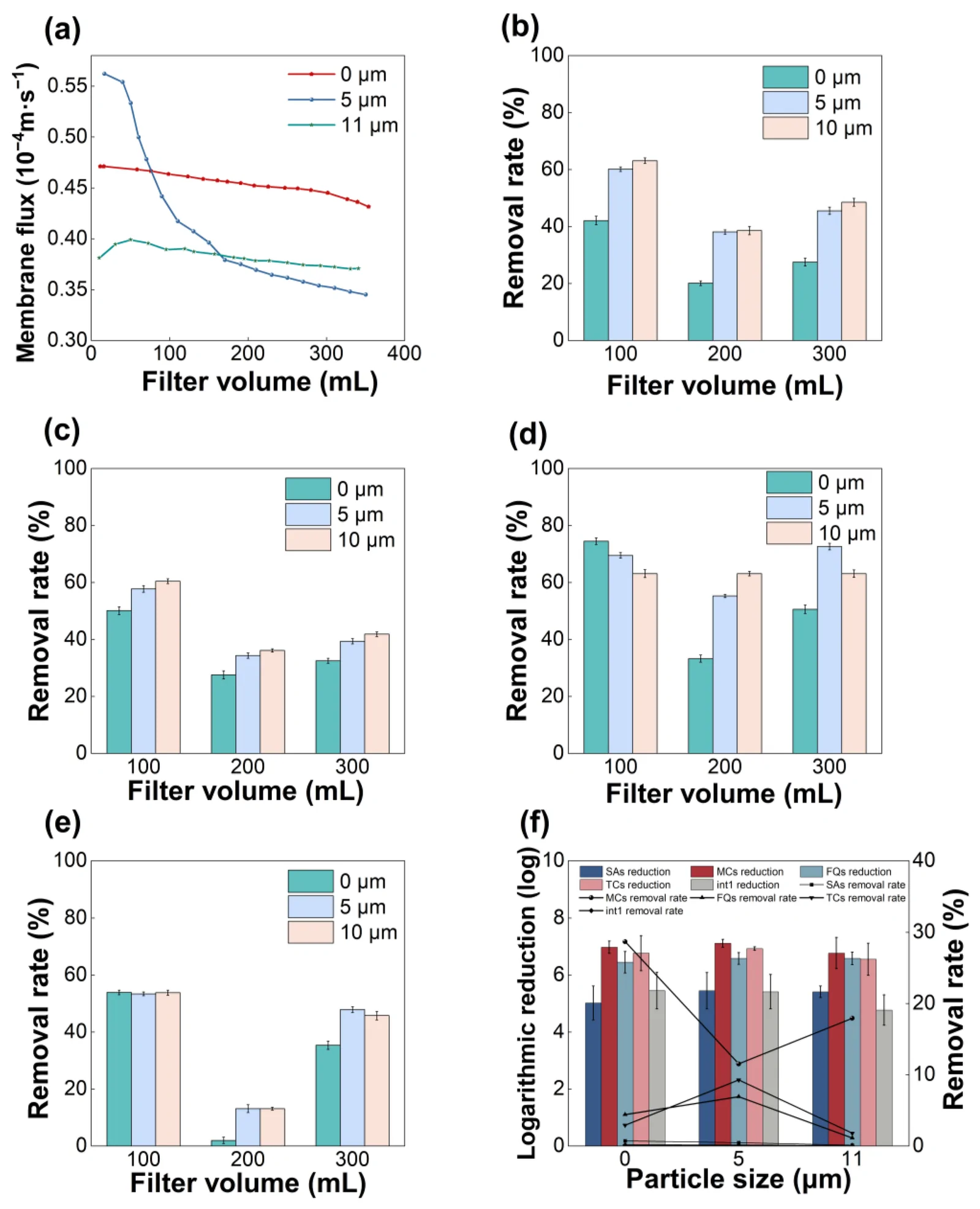
Membrane-flux variation and contaminant reduction by PES/PVA/TiO_2_-100 under different particulate-matter conditions: (**a**) membrane flux; (**b**) SMX; (**c**) ROX; (**d**) TC; (**e**) OFL; (**f**) ARGs.

The membrane-flux results (Figure 5a) showed that the 5 μm particle group exhibited the fastest flux decline and the lowest final flux, whereas the flux of the 10 μm particle group was lower than that of the particle-free group but changed relatively slowly in the later stage. This indicates that smaller particles, with larger specific surface areas and more deposition contact points, were more likely to form a dense particle layer on the membrane surface, thereby aggravating membrane fouling and filtration resistance [28].

The antibiotic removal results (Figure 5b–e) showed that the coexistence of particulate matter generally improved antibiotic removal efficiencies. After the addition of 5 μm particles, the removal efficiencies of SMX, ROX, TC, and OFL increased by 27.0%, 6.2%, 22.0%, and 12.5%, respectively, with SMX and TC showing the most pronounced responses. Because SMX was weakly retained in the absence of particles, the particle-deposition layer and additional adsorption sites formed by particulate matter markedly improved its apparent removal efficiency. Suspended particles can affect the adsorption and migration behavior of SMX. The pronounced response of TC may be related to its molecular structure. TC contains multiple hydroxyl, carbonyl, and nitrogen-containing functional groups, which can readily interact with hydroxyl groups, metal oxide sites on particle surfaces, or polar groups on the membrane surface through hydrogen bonding, complexation, and electrostatic interactions. Therefore, TC was more easily removed through the combined effects of particle adsorption and membrane retention [46]. In contrast, ROX and OFL were relatively less affected by particulate matter in this system, indicating that the enhancement effect of particles was contaminant-selective.

The stage-dependent variations in apparent antibiotic retention under particulate-matter conditions are consistent with the evolving combined effects of particle-associated antibiotic retention, membrane–solute interactions, and increasing filtration resistance during sequential filtration. These variations are therefore discussed as an overall dynamic response rather than being assigned to a single particle-related process.

The ARG reduction results (Figure 5f) showed that particulate matter did not produce a universal promoting effect on ARG removal. In the 5 μm particle group, FQs-ARGs and TCs-ARGs increased only slightly, whereas MCs-ARGs decreased. In the 10 μm particle group, all ARG classes except MCs-ARGs decreased. This indicates that ARG removal depends not only on the particle-deposition layer, but also on DNA occurrence forms, carrier size, and the binding state of ARGs with particles or the membrane surface [47]. Overall, particulate matter enhanced the removal of some antibiotics, especially SMX and TC, but also aggravated membrane fouling. ARGs still showed class-dependent differences and limited reductions.

### 3.4. Membrane Fouling Characteristics

Membrane fouling behavior was evaluated by integrating flux decline, post-filtration FTIR-ATR spectral changes, and Hermia-model fitting. The best-fitting Hermia model was used to identify the predominant hydraulic fouling pattern, while the corresponding FTIR-ATR changes provided complementary information on the exposure and masking of the characteristic membrane-surface functional groups.

**Figure 6:**
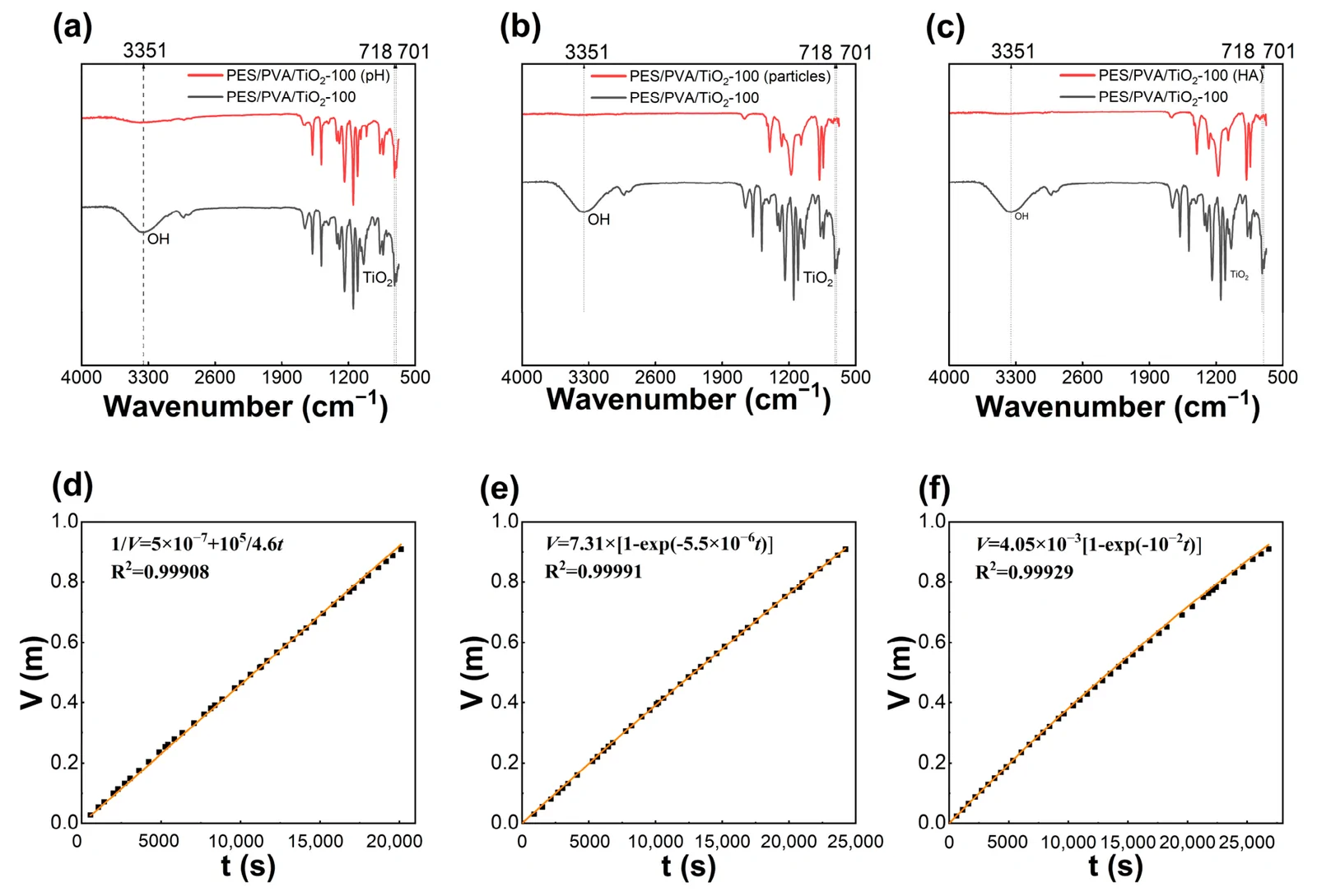
FTIR-ATR spectra of fouled membrane surfaces and Hermia model fitting results: (**a**) FTIR-ATR spectra before and after filtration in the pH experiment; (**b**) FTIR-ATR spectra before and after filtration in the particulate-matter experiment; (**c**) FTIR-ATR spectra before and after filtration in the HA experiment; (**d**) Hermia model fitting under different pH conditions; (**e**) Hermia model fitting under particulate-matter conditions; (**f**) Hermia model fitting under HA conditions.

Figure 6 compares the diagnostic O–H and Ti–O spectral markers identified in Section 3.1 before and after filtration. Under the pH-regulated conditions without added HA or particulate matter (Figure 6a), the O–H band at 3150–3650 cm^−1^ and the Ti–O bands at 718 and 701 cm^−1^ remained distinguishable after filtration, although their intensities decreased. This indicates that the composite layer remained spectroscopically exposed rather than being completely masked by continuous surface deposition. Together with the relatively gradual flux decline and the best fit to the standard pore-blocking model (*R*^2^ = 0.99908; Figure 6d), these observations support pore-associated filtration resistance as the dominant hydraulic fouling characteristic under these conditions rather than extensive surface coverage.

In contrast, under HA and particulate-matter conditions (Figure 6b,c), the O–H and Ti–O bands were markedly attenuated or masked after filtration, suggesting greater masking of the PVA/chitosan/TiO_2_ surface by accumulated foulants. This spectral response was consistent with the more pronounced flux decline described in Section 3.3. The filtration data under the HA and particulate-matter conditions were best fitted by the complete-blocking model, with *R*^2^ values of 0.99929 and 0.99991, respectively (Figure 6e,f). The consistency among flux decline, FTIR-ATR spectral masking, and Hermia-model fitting suggests predominantly complete-blocking-type hydraulic behavior accompanied by increased foulant accumulation at the membrane interface.

Overall, the water matrix affected the predominant fouling behavior of PES/PVA/TiO_2_-100. Under the tested pH conditions without added HA or particles, the flux data were better described by the standard-blocking model, and the characteristic membrane-surface bands remained distinguishable after filtration. In the presence of HA or particulate matter, the flux data were better described by the complete-blocking model and were accompanied by greater attenuation of the characteristic FTIR-ATR bands. These results suggest a shift from predominantly pore-associated hydraulic resistance to a fouling response involving greater foulant accumulation at the membrane interface.

### 3.5. Relationship Between Removal Performance and Membrane Fouling

The principal role of the PVA/chitosan hydrogel coating combined with in situ TiO_2_ incorporation was to create a more functional composite interface while maintaining the basic hydraulic characteristics of the PES ultrafiltration membrane. Functionalized PES-based membranes have also been reported to enhance antibiotic retention through interfacial modification; this is consistent with the conclusion of Prem Gaudel et al. [50,51]. Because the molecular weights of all four target antibiotics are far below the 100 kDa MWCO, the enhanced retention after modification cannot be attributed primarily to size exclusion and is more reasonably associated with the strengthened membrane–antibiotic interfacial interactions provided by the PVA/chitosan/TiO_2_ composite layer [23,24,40]. Thus, for low-molecular-weight antibiotics, the functionalized interface provides additional retention beyond conventional UF size exclusion.

The antibiotic data reported in this study represent stage-resolved apparent retention at cumulative filtrate volumes of 100, 200, and 300 mL rather than cumulative removal determined from a complete mass balance. All samples were subjected to the same filtration, dilution, and analytical procedures, and the diluted antibiotic concentrations were within the validated analytical range. The non-monotonic variations among sampling stages are therefore discussed as stage-dependent responses of the evolving filtration system. The interpretation of antibiotic-retention behavior is based on the overall consistency among membrane modification, water-matrix conditions, stage-resolved permeate concentrations, flux behavior, FTIR-ATR spectral changes, and Hermia-model fitting rather than on any individual stage-to-stage fluctuation.

As shown in Section 3.3 and Section 3.4, pH mainly affected apparent antibiotic retention by altering antibiotic ionization and membrane–solute interactions. The HA-associated response may reflect the combined contributions of size-dependent retention of HA-associated components, HA–antibiotic interactions, and HA accumulation at the membrane interface. Particulate matter may provide additional adsorption and interfacial-accumulation effects [19,20]. Both HA and particulate matter were accompanied by increased filtration resistance, indicating a trade-off between apparent antibiotic retention and hydraulic performance under complex water-matrix conditions. The key response under complex water-matrix conditions was therefore a trade-off between enhanced antibiotic retention and fouling development rather than a simple improvement in either retention or antifouling performance.

Compared with the antibiotics, membrane-associated retention of the standard plasmid ARGs responded less strongly to composite modification and remained target dependent. The ARG results therefore primarily describe the influence of PVA/chitosan/TiO_2_ modification on plasmid-DNA accumulation and retention at the membrane interface and are not extended to the overall removal of environmentally occurring ARGs.

Table S2 further clarifies the characteristics of the present membrane system [8,52,53,54,55]. In contrast to several recently reported electroactive, photocatalytic, or biologically coupled membrane processes, PES/PVA/TiO_2_-100 operates under conventional pressure-driven ultrafiltration without additional electrical or light assistance. Meanwhile, the present study evaluates multiple antibiotic classes and ARGs together with the effects of pH, humic acid, particulate matter, and membrane fouling. These comparisons better demonstrate the performance characteristics and technological positioning of the developed membrane.

## 4. Conclusions

This study developed a PES/PVA/TiO_2_-100 modified ultrafiltration membrane by combining PVA/chitosan hydrogel coating with in situ TiO_2_ incorporation and systematically evaluated its antibiotic and ARG removal and fouling behavior under different pH, HA, and particulate-matter conditions. The results demonstrate that the composite modification not only improved the interfacial properties and antibiotic-retention performance of the PES membrane, but also influenced the relationship between contaminant retention and membrane fouling under different water-quality conditions. The main conclusions are as follows:

This study prepared a PES/PVA/TiO_2_-100 modified membrane and evaluated its removal of typical antibiotics and ARGs, as well as membrane fouling, under different pH, HA, and particulate-matter conditions. PVA/chitosan hydrogel coating combined with in situ TiO_2_ embedding improved PES membrane hydrophilicity and hydraulic performance, enhanced antibiotic removal, but also revealed a trade-off between removal enhancement and fouling aggravation in complex water matrices. The main conclusions are as follows:

PES/PVA/TiO_2_-100 formed an approximately 5 μm gel layer with O–H and Ti–O characteristic peaks. Its Ra decreased from 6.946 to 2.344 nm, static water contact angle from 94.5° to 67.2°, and pure-water flux increased by approximately 5.0%, confirming improved surface hydrophilicity and baseline hydraulic performance.

Compared with PES-100, PES/PVA/TiO_2_-100 showed an approximately 4.9% higher final flux and increased SMX, ROX, TC, and OFL removal efficiencies by more than 10%, with the greatest improvement for OFL, at approximately 14%. ARG reduction also increased on average, but remained class-dependent, indicating stronger enhancement for typical antibiotics than for ARGs.

Water-quality conditions strongly affected removal performance. Low pH favored SMX, TC, and OFL removal; 5 mg·L^−1^ HA increased TC removal by 16.8%; and 5 μm particles increased SMX and TC removal by 27.0% and 22.0%, respectively. In contrast, HA and particles did not universally promote ARG reduction.

Integrated analysis of flux decline, FTIR-ATR spectral changes, and Hermia-model fitting suggested predominantly standard-blocking-type hydraulic behavior under the tested pH conditions. In the presence of HA or particles, the flux data were better fitted by the complete-blocking model and were accompanied by greater masking of the characteristic membrane-surface bands, suggesting increased foulant accumulation at the membrane interface. Overall, the PVA/chitosan/TiO_2_ composite modification improved the interfacial properties and antibiotic-retention performance of the PES ultrafiltration membrane. The results demonstrated that water-matrix composition strongly influenced the balance between contaminant retention and filtration resistance.

## Figures and Tables

**Table 1:** Primer information for target and reference genes.

Gene Class	Target Gene	Primer Sequence (5′–3′)	Amplicon Length (bp)	Tm (°C)
Quinolone resistance genes	*qnrS*	F: GTGAGTAATCGTATGTACTTTTGC	169	58
		R: AAACACCTCGACTTAAGTCT		
	*qnrD*	F: GGAGCTGATTTTCGAGGG	105	62
		R: AGAAAAATTAGCGTAACTAAGATTTGTC		
Sulfonamide resistance genes	*sul1*	F: CACCGGAAACATCGCTGCA	158	58
		R: AAGTTCCGCCGCAAGGCT		
	*sul2*	F: GTCAAAGAACGCCGCAATGT	105	58
		R: TCATCTGCCAAACTCGTCGTTA		
Tetracycline resistance genes	*tetM*	F: CATCATAGACACGCCAGGACATAT	101	58
		R: CGCCATCTTTTGCAGAAATCA		
	*tetO*	F: CAACATTAACGGAAAGTTTATTGTATACCA	104	60
		R: TTGACGCTCCAAATTCATTGTATC		
	*tetW*	F: ATGAACATTCCCACCGTTATCTTT	101	60
		R: ATATCGGCGGAGAGCTTATCC		
Macrolide resistance genes	*ermA*	F: TTGAGAAGGGATTTGCGAAAAG	76	60
		R: ATATCCATCTCCACCATTAATAGTAAACC		
	*ermB*	F: TAAAGGGCATTTAACGACGAAACT	172	62
		R: TTTATACCTCTGTTTGTTAGGGAATTGAA		
Class 1 integron	*intI* *1*	F: CGAACGAGTGGCGGAGGGTG	312	58
		R: *intI* TACCCGAGAGCTTGGCACCCA		
Housekeeping gene	*16S rRNA gene*	F: GGGTTGCGCTCGTTGC	60	58
		R: ATGGYTGTCGTCAGCTCGTG		

**Table 2:** Key physicochemical parameters of PES-100 and PES/PVA/TiO_2_-100.

Parameter	PES-100	PES/PVA/TiO_2_-100	Change
Rmax (nm)	20.486	7.808	Decreased
Ra (nm)	6.946	2.344	Decreased
Rms (nm)	2.208	0.873	Decreased
Static water contact angle (°)	94.5	67.2	Decreased
Pure-water flux (m·s^−1^)	2.16 × 10^−4^	2.26 × 10^−4^	Increased by approximately 5.0%
Characteristic peaks	PES characteristic peaks	New O–H and Ti–O peaks	Introduction of PVA/TiO_2_

## Data Availability

The original contributions presented in this study are included in the article. Further inquiries can be directed to the corresponding authors.

## Supplementary Materials

The following supporting information can be downloaded at: https://www.mdpi.com/article/10.3390/membranes16090293/s1, Figure S1: Schematic diagram of the laboratory-scale constant-pressure ultrafiltration system used in this study; Table S1: Operating conditions of the laboratory-scale ultrafiltration experiments; Table S2: Comparison of the developed PES/PVA/TiO_2_-100 membrane with selected recent membrane systems for antibiotic and antibiotic-resistance control.

## Abbreviations

The following abbreviations are used in this manuscript:

PES	Polyethersulfone
PVA	Polyvinyl alcohol
UF	Ultrafiltration
ARGs	Antibiotic resistance genes
SMX	Sulfamethoxazole
ROX	Roxithromycin
TC	Tetracycline
OFL	Ofloxacin
HA	Humic acid
UPLC-MS/MS	Ultra-performance liquid chromatography-tandem mass spectrometry
ESI+	Positive-ion electrospray ionization
MRM	Multiple reaction monitoring
qPCR	Real-time quantitative polymerase chain reaction
16S rDNA	16S ribosomal DNA
SEM	Scanning electron microscopy
AFM	Atomic force microscopy
FTIR-ATR	Attenuated total reflection Fourier transform infrared spectroscopy
BET	Brunauer–Emmett–Teller
Rmax	Maximum height difference
Ra	Average roughness
Rms	Root-mean-square roughness
SAs-ARGs	Sulfonamide resistance genes
MCs-ARGs	Macrolide resistance genes
FQs-ARGs	Fluoroquinolone resistance genes
TCs-ARGs	Tetracycline resistance genes
