# Supplementary material for: Effects of PVA/TiO2 Composite Hydrogel-Modified PES Ultrafiltration Membranes on Antibiotic Removal Performance and Membrane Fouling Behavior
Source: Membranes (Basel). 2026 Aug 31;16(9):293. doi: 10.3390/membranes16090293 (PMC13608905; doi:10.3390/membranes16090293)
Supplement: Supplementary file 1 [file membranes-16-00293-s001.zip › membranes-4479481-supplementary.pdf]

Effects of PVA/TiO_2 Composite Hydrogel-Modified PES Ultrafiltration Membranes on Antibiotic Removal Performance and Membrane Fouling Behavior

List of Supplementary Materials

Table S1. Operating conditions of the laboratory-scale ultrafiltration experiments.

Table S2. Comparison of the developed PES/PVA/TiO_2-100 membrane with selected recent membrane systems for antibiotic and antibiotic-resistance control.

Figure S1. Schematic diagram of the laboratory-scale constant-pressure ultrafiltration system.

Table S1. Operating conditions of the laboratory-scale ultrafiltration experiments.

**Parameter**	**Operating condition**
Ultrafiltration cell volume	400 mL
Feed volume	400 mL
Effective membrane area	38.4 cm^2
Dead volume	~1 mL
Operating pressure	0.10 MPa
Stirring speed	200 rpm
Cumulative filtrate volume for sampling	100, 200, and 300 mL
Sample volume at each sampling point	50 mL

Table S2. Comparison of the developed PES/PVA/TiO_2-100 membrane with selected recent membrane systems for antibiotic and antibiotic-resistance control.

**Membrane/process**	**Target contaminants**	**Driving/assistance mode**	**Evaluation scope**	**Representative performance**
Conventional UF	7 antibiotics, ARB, ARGs	Pressure-driven UF	Antibiotics+ antimicrobial resistance	Antibiotics: 19-95%; ARG removal: >99% [52]
Electroactive C/PVDF UF	SMX, ARB, ARGs	Electrically assisted UF	Antibiotic+ARB+ARGs +fouling	SMX degradation: 81.5%; normalized flux: +30.4% at +4 V [8]
Photocatalytic PVDF/TM UF	Antibiotics, bacteria, ARGs	Visible-light-assisted UF	Multi-contaminant removal+photocatalysis	Antibiotics/bacteria/ARGs: 86.5/91.4/91.8% [53]
BisBAL-modified UF	CPFX and micropollutants	Functionalized UF	Real hospital wastewater +biofouling	Optimized HF membrane: >80% CPFX rejection [54]
Iron-tannin functionalized UF	TC	Gravity-driven; biological coupling	TC removal+effluent quality+fouling	Effluent TC: 0.38 mg·L^{-1} [55]
PES/PVA/TiO_2-100 (this study)	SMX, ROX, TC, OFL, ARGs	Conventional pressure-driven UF (0.10 MPa); no electrical/light assistance	Antibiotics+ARGs+pH+ HA+particles+fouling	Compared with PES-100, removal of all four antibiotics increased by >10%; the greatest increase was approximately 14% for OFL

[52] Michael, S. G.; Drigo, B.; Michael-Kordatou, I.; Michael, C.; Jäger, T.; Aleer, S. C.; Schwartz, T.; Donner, E.; Fatta-Kassinos, D. The effect of ultrafiltration process on the fate of antibiotic-related microcontaminants, pathogenic microbes, and toxicity in urban wastewater. *Journal of Hazardous Materials* **2022**, *435*, 128943. DOI: 10.1016/j.jhazmat.2022.128943.

[8] Li, J.; Ren, S.; Qiu, X.; Zhao, S.; Wang, R.; Wang, Y. Electroactive ultrafiltration membrane for simultaneous removal of antibiotic, antibiotic resistant bacteria, and antibiotic resistance genes from wastewater effluent. *Environmental Science & Technology* **2022**, *56*, 15120–15129. DOI: 10.1021/acs.est.2c00268.

[53] Li, R.; Wei, Z.; Li, P.; Qiu, Y.; Wang, C.; Wang, C.; Ren, L.-F.; Shao, J.; He, Y. Novel visible-light activated photocatalytic ultrafiltration membrane for simultaneous separation and degradation of emerging contaminants. Journal of Hazardous Materials **2024**, 478, 135634. DOI: 10.1016/j.jhazmat.2024.135634.

[54] Kose-Mutlu, B.; Ilyasoglu, G.; Ardic, R.; Demirbilekli, M. A.; Koyuncu, I. Removal of emerging organic micropollutants from real hospital wastewater by modified ultrafiltration membranes. Separation and Purification Technology **2025**, 357, 129960. DOI: 10.1016/j.seppur.2024.129960.

[55] Xia, T.; Yang, Y.; Yang, Z.; Qi, J.; Zhu, Z.; Zhou, Y.; Li, J. Synergistic enhancement of tetracycline removal and membrane fouling mitigation in anaerobic gravity-driven bioreactors using iron-tannin framework functionalized ultrafiltration membranes. Separation and Purification Technology **2026**, 388, 136381. DOI: 10.1016/j.seppur.2025.136381.

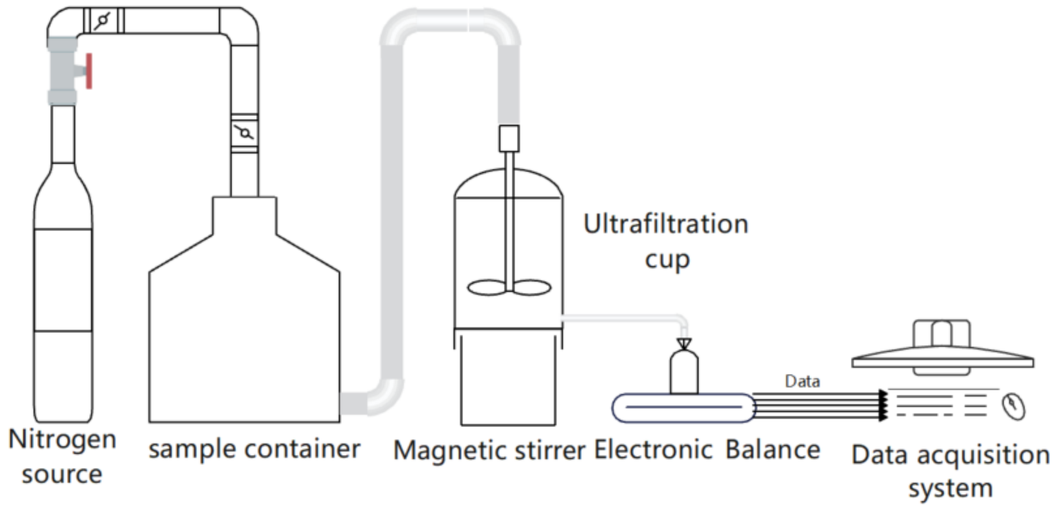

Figure S1. Schematic diagram of the laboratory-scale constant-pressure ultrafiltration system used in this study.
